# Evolution of Survival in Patients with Multiple Myeloma over Two Decades: A Real-World Experience from a Medium-Level Hospital

**DOI:** 10.3390/cancers17050793

**Published:** 2025-02-25

**Authors:** Esther Ortega-Vida, Abel Rosado-Rodriguez, Rocio Fe, Victoria Verdugo, Rocio Gavira, Sebastián Garzón

**Affiliations:** 1 Department of Hematology, University Hospital of Jerez, 11407 Jerez de la Frontera, Spain; abelrosadorodriguez@gmail.com (A.R.-R.); rocio.fe.sspa@juntadeandalucia.es (R.F.); mariav.verdugo.sspa@juntadeandalucia.es (V.V.); sebastianf.garzon.sspa@juntadeandalucia.es (S.G.); 2 Department of Pharmacy, University Hospital of Jerez, 11407 Jerez de la Frontera, Spain

**Keywords:** multiple myeloma, novel therapies, survival, real-world data

## Abstract

Background: The gradual introduction of numerous therapeutic advancements over recent years in the treatment of patients with multiple myeloma (MM) appears to have contributed to significant improvements in overall survival (OS). Methods: We conducted a single-center retrospective observational study, including all MM patients treated at the University Hospital of Jerez de la Frontera, diagnosed between 1 January 2000, and 31 December 2022. Patients were divided into three calendar periods (2000–2007, 2008–2015, and 2016–2022) and two patient groups (candidates and non-candidates for autologous hematopoietic progenitor transplantation). Results: A total of 420 myeloma patients were included in this study, with a median age of 64 years. The median survival steadily improved from 50.7 months (33.8–73.2; 95% CI) in 2000–2007 to 72.4 months (57.5–98.2; 95% CI) in 2008–2015 and has not yet been determined in the 2016–2022 cohort (*p* = 0.008). OS improved in all age groups, even in older patients. The median OS in patients not undergoing autologous stem cell transplantation (ASCT) was 38.8 months (31.5–51.6; 95% CI) compared with 132.66 months (110.5–150.9; 95% CI) in those who underwent this procedure (*p* < 0.0001). The introduction of novel drug classes in first-line treatment significantly improved OS compared with traditional chemotherapy (56.7 months). The median OS increased to 78.8 months (95% CI: 68.8–113.3; *p* = 0.03) with proteasome inhibitors (PIs), 99.4 months (95% CI: 99.4–NA; < 0.0001) with immunomodulatory drugs (IMIDs), and was not determined for anti-CD38 monoclonal antibodies (*p* = 0.02). Conclusions: Survival outcomes for MM have significantly improved over the past two decades, particularly among younger and ASCT-eligible patients. However, according to all studies, disparities persist across healthcare settings, underscoring the need for equitable access to modern therapies and optimized management strategies.

## Simple Summary

Multiple myeloma is a cancer of plasma cells that has seen remarkable improvements in treatment outcomes over the last few years. Advances such as proteasome inhibitors, immunomodulatory drugs, and monoclonal antibodies have significantly extended survival, especially when these therapies are introduced early in the disease course. Our study, conducted in a real-world setting at a mid-level hospital in Spain, analyzed 420 multiple myeloma cases treated over 22 years. The results reflect clinical practice as being more closely aligned with everyday healthcare and show steady increases in survival rates, particularly among younger patients and those eligible for stem cell transplantation. These findings highlight the importance of ensuring timely access to modern therapies and optimizing treatment strategies to improve outcomes for all patients.

## 1. Introduction

Multiple myeloma (MM), the second most common hematologic malignancy, is characterized by the clonal expansion of plasma cells in the bone marrow and the production of monoclonal immunoglobulin. It constitutes about 15% of annual reported cases of hematological malignancies in the Western world [1]. Despite significant advances in MM treatment, including proteasome inhibitors (PIs), immunomodulatory agents (IMIDs), and monoclonal antibodies (MoABs), the disease remains incurable. In 2020, MM accounted for an estimated 117,077 deaths globally and 2108 in Spain [2,3,4].

Although the incidence and mortality rates of MM show a great variation around the world, several studies have reported a dramatic increase in its incidence in the second half of the 20th century and continuing into this century, with more than doubled global cases from 1990 to 2019 [5,6,7,8,9,10,11,12,13,14]. SEER estimated an increase in the annual incidence from 5.5 to 6.8 cases per 100,000 persons in 1992 and 2021, respectively [15]. In Spain, the incidence calculated was 2729 cases in 2016 and 3214 in 2020, with a higher incidence in older age groups (851 cases occurred in individuals under 65 years, 868 in those aged 65–75 years, and 1495 in individuals over 75 years) [16,17].

Over the past two decades, several epidemiological studies have demonstrated significant improvements in survival, largely attributed to the introduction of autologous stem cell transplantation (ASCT) and novel therapeutic agents [18,19,20,21,22,23,24,25,26,27,28,29]. For instance, a population-based study from the provinces of Girona and Granada reported an increase in 5-year survival from 27.4% in 1994 to 47.4% in 2016 among 1957 MM patients [30]. However, the majority of survival data in the literature originate from highly specialized centers or clinical trials, where patients typically have earlier access to state-of-the-art therapies compared with the broader patient population worldwide. In these specialized settings, recent studies from Spanish centers have reported OS rates ranging from 86.7 to 103.6 months [31,32,33].

In contrast, the present study examined real-world outcomes in a medium-level hospital, offering critical insights into the implementation and efficacy of modern therapeutic strategies in a more representative patient population. This approach reflects the clinical realities faced by most MM patients worldwide and aligns more closely with their expectations and experiences.

## 2. Materials and Methods

This retrospective, observational, single-center study was conducted at Jerez University Hospital, a medium-level hospital representative of typical Spanish clinical practice. This study included all patients diagnosed and/or treated for symptomatic MM at the hospital, as well as those referred for ASCT between 1 January 2000, and 31 December 2022. Patients were divided into three cohorts based on the year of diagnosis: 2000–2007 (cohort A), 2008–2015 (cohort B), and 2016–2022 (cohort C). Further stratification was performed according to ASCT eligibility into transplant candidates and non-candidates. The follow-up cut-off date was 15 November 2023.

Data were obtained from the hospital’s monoclonal gammopathies registry and clinical records. OS was defined as the time from diagnosis to death or the last follow-up. Survival analysis was conducted using the Kaplan–Meier method, and differences between groups were assessed with the log-rank test. Multivariable analysis was performed using a Cox proportional hazards regression model to estimate hazard ratios (HRs), with verification of the proportional hazards assumption.

The impact of incorporating novel therapeutic agents in first-line treatment—such as PIs, IMiDs, and MoABs—either as monotherapy or in combination, was analyzed and compared with standard chemotherapy regimens.

This study received approval from the hospital’s ethics committee, and all procedures adhered to ethical research guidelines, including the Declaration of Helsinki.

## 3. Results

**Figure 1:**
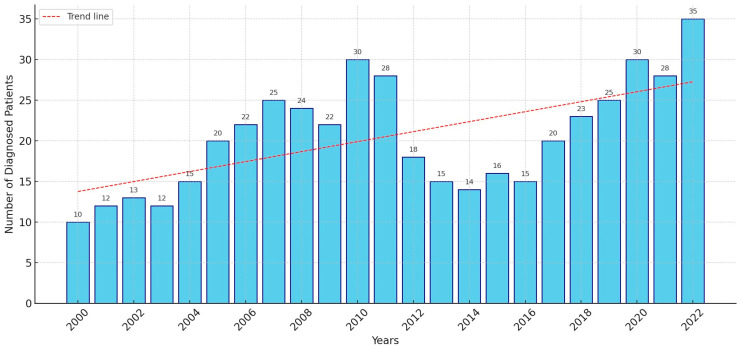
Evolution of disease incidence at our hospital over time.

Among the 420 patients included in this study, 107 were in cohort A, 149 in cohort B, and 164 in cohort C (Table 1). The average annual incidence was 18.3 cases (range: 9–26), with 13.4 cases (range: 9–22) in cohort A (2000–2007), 18.6 cases (range 15–28) in cohort B (2008–2015), and 23.4 cases (range 13–36) in cohort C (2016–2022) (Figure 1). 

The induction treatment regimen evolved significantly over the study period, particularly in recent years. In cohort A (2000–2007), 95.3% of patients received standard chemotherapy, while only 1.8% and 2.8% were treated with PIs and IMiDs, respectively. In cohort B (2008–2015), the proportion of patients receiving chemotherapy decreased to 27.5%, with PIs and IMiDs administered to 68.4% and 13.4% of patients, respectively. By cohort C (2016–2022), chemotherapy was used exclusively for palliative purposes in 6.7% of patients, while the use of PIs and IMiDs increased to 86.6% and 70.1%, respectively. Additionally, 20.1% of patients in this cohort received anti-CD38 monoclonal antibodies as part of their first-line treatment.

**Figure 2:**
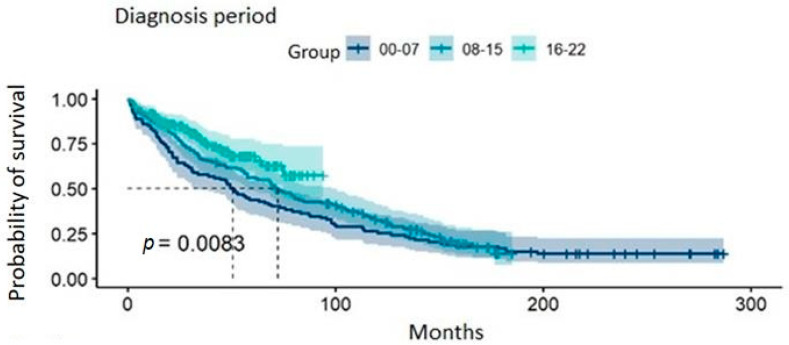
Graphical representation of the Kaplan–Meier estimator for overall survival by patient groups according to the diagnosis period variable. Dashed lines indicate the median of each group, and shaded areas correspond to the 95% confidence intervals. The *p*-value corresponds to the result of the log-rank test.

The median follow-up time from diagnosis to the end of the follow-up period for the global population was 130 months (IQR: 53.4–192.0), with 232.0 months (IQR: 205.3–260.65) in cohort A, 148.1 months (IQR: 121.38–171.49) in cohort B, and 45.5 months (IQR: 25.25–66.75) in cohort C. The median OS improved significantly over time. For the entire cohort, the median OS increased from 50.7 months (95% CI: 33.9–73.2) in cohort A to 72.5 months (95% CI: 57.5–98.2) in cohort B (*p* = 0.0083). In cohort C, the median OS had not been reached at the time of analysis (Figure 2). The 48-month survival rate was 52.3% in the first cohort, increasing to 61.9% in the second cohort and finally reaching 71.4% in the third cohort.

**Figure 3:**
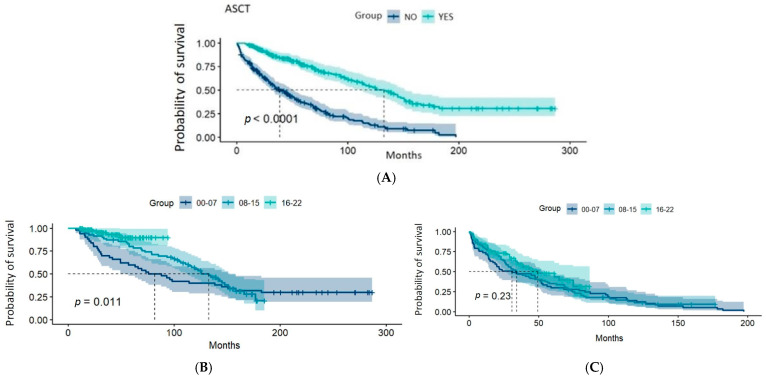
Graphical representations of the Kaplan–Meier estimator for overall survival by patient groups based on whether they underwent autologous hematopoietic progenitor cell transplantation: (**A**) all patients; (**B**) patients who underwent transplantation by time periods; (**C**) patients who did not undergo transplantation by time periods. The dashed lines indicate the medians of each group, and the shaded areas correspond to the 95% confidence intervals. The *p*-value corresponds to the result of the log-rank test.

The median OS in patients who did not undergo auto-HSCT was 38.81 months (31.57–51.61; 95% CI) compared with 132.66 months (110.59–150.98; 95% CI) in those who did undergo this procedure (*p* < 0.0001). In patients eligible for ASCT, the median OS improved from 81.8 months (95% CI: 49.3–158.0) in cohort A to 132.7 months (95% CI: 110.6–152.0) in cohort B, with the median OS still not reached in cohort C (*p* = 0.011). Conversely, among non-transplant candidates, the increase in OS was less pronounced, rising from 30.8 months (95% CI: 17.0–52.1) in cohort A to 49.3 months (95% CI: 35.1–NA) in cohort C (*p* = 0.23) (Figure 3).

As anticipated, improvements in OS were more pronounced in younger patients. In patients under 60 years, the median OS increased significantly from 87.7 months in cohort A to 125.4 months in cohort B, with the median OS not yet reached in cohort C (*p* = 0.10). Among those aged 60–70 years, the median OS rose from 49.2 months in cohort A to 83.8 months in cohort B, with the median OS not yet reached in cohort C (*p* = 0.03). In patients over 70 years, the OS gains were comparatively smaller, increasing from 17.0 months in cohort A to 32.5 months in cohort B and 49.2 months in cohort C (*p* = 0.03).

**Figure 4:**
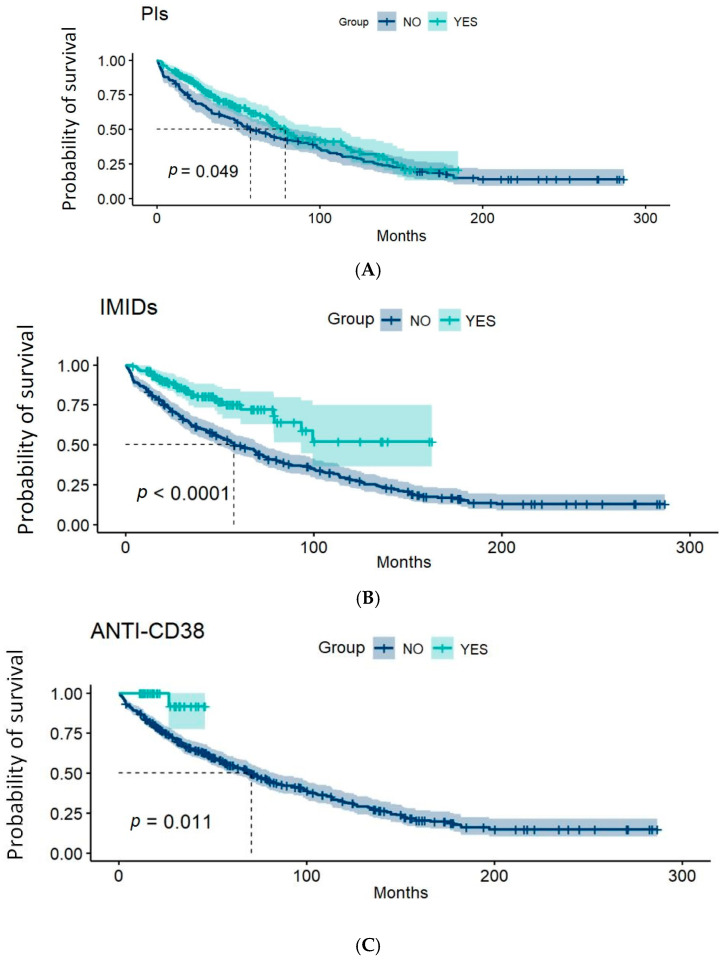
Graphical representations of the Kaplan–Meier estimator for overall survival by patient groups according to the therapy received: (**A**) patients who incorporated PIs in their treatments; (**B**) patients who incorporated IMIDs in their treatments; (**C**) patients receiving anti-CD38 monoclonal antibodies. The dashed lines indicate the medians of each group, and the shaded areas correspond to the 95% confidence intervals. The *p*-value corresponds to the result of the log-rank test.

We further assessed the impact of each new drug class on overall survival (OS) by comparing it with the median OS achieved with traditional chemotherapy, which was 56.7 months (95% CI: 44.4–79.0). Patients who incorporated PIs in their treatments had a median OS of 78.8 months (95% CI: 68.8–113.3; *p* < 0.05). In the group that incorporated immunomodulatory drugs, the median OS had not been reached at the time of analysis (*p* < 0.0001). Similarly, for patients receiving anti-CD38 monoclonal antibodies, the median OS had not been reached at the time of analysis (*p* = 0.011) (Figure 4).

Finally, we analyzed the OS improvement following the introduction of each drug class. The first patient treated with a PI was diagnosed in October 2007. The median OS for the 112 patients diagnosed prior to this was 51.8 months (95% CI: 41.6–75.6), compared with 78.9 months (95% CI: 69.1–104.5) for those treated after (*p* = 0.079). Similarly, the first patient treated with an IMID (thalidomide) was diagnosed in December 2006. The median OS increased from 56.7 months (95% CI: 47.1–87.8) in the 83 patients diagnosed earlier to 75.9 months (95% CI: 68.1–98.3) thereafter (*p* = 0.39). For anti-CD38 monoclonal antibodies, the first patient treated during induction was diagnosed in January 2020, with a median OS of 68.5 months (95% CI: 56.1–80.1) before their introduction, while the median OS was not reached after (*p* < 0.01).

## 4. Discussion

This retrospective study confirms a progressive and significant improvement in the OS of MM patients over the past two decades, particularly among younger, transplant-eligible patients. To our knowledge, this is the first long-term global survival analysis in MM conducted at an intermediate-level hospital, representative of routine clinical practice in average hospital settings in many countries. This distinguishes our findings from prior studies performed in highly specialized centers with rapid access to novel therapies and advanced diagnostic and supportive resources.

In our country, four key studies have investigated survival trends in MM. Chang-Chan et al. analyzed 1957 patients from epidemiological registries in Girona and Granada (1994–2016) and observed an increase in five-year survival from 27.4% to 47.4% across three time periods, with the greatest improvements in younger patients (15–49 years) and minimal changes in those over 70 [30]. Rodríguez-Lobato et al. reported median overall survival (OS) improvements across three cohorts (1970–2015) at the Hospital Clínic of Barcelona, noting a median OS of 61.2 months in the 2000–2015 cohort, with significant gains observed in patients under 65 years of age (*p* < 0.001) [31]. Similarly, López-Muñoz et al. evaluated survival trends across 20 years in Hospital 12 de Octubre, showing a median OS increase from 41.4 months (1999–2003) to 86.7 months (2016–2019) for patients under 75 (*p* = 0.001), while the OS for older patients remained static at 31.1 months [32]. Finally, Puertas et al. at Salamanca University Hospital highlighted significant survival improvements over four decades (22.4 months in 1980–1990 to 103.6 months in 2011–2020; *p* < 0.001), with the greatest gains observed following the introduction of novel therapies [33].

International studies echo these findings. Nunnelee et al. (Ohio State University) demonstrated an increased three-year OS among ASCT recipients (from 45% to 80%). While progression-free survival improved significantly in patients over 65 years (*p* < 0.01), no significant OS improvements were observed in this group (*p* = 0.054) [20]. Blimark et al. [22] reported a five-year OS increase in Swedish and Danish registries (2005–2018), from 59.8% to 73.4% in patients ≤ 65 years in Denmark and from 27.5% to 39.8% in patients >65 years in Sweden [21]. Similarly, Tang et al. [26], using data from Taiwan’s NHIRD (2007–2015), reported a median OS increase from 2.1 years to 3.12 years across two time periods [25].

Despite improvements in OS, significant differences persist across regions. For example, the OS was as high as 103.2 months at Hospital de Salamanca during the period of 2011–2020 compared with only 3.12 years (approximately 37 months) in Taiwan during 2013–2015. Similarly, the 5-year OS was 47.4% in the study by Chang et al. (2010–2016) and 73.4% in the study by Blimark et al. (2014–2019). These disparities may be explained by systemic barriers limiting access to innovative treatments, such as differences in national healthcare policies, delays in drug approval, and discrepancies in hospital infrastructure and resources. The differences between countries are not only concerning but also difficult to overcome, much like many other aspects related to the social protection available to citizens in each region. However, each healthcare center, particularly medium- and secondary-level hospitals, must be aware of the importance of timely access to innovation. This can be achieved by promoting conducting clinical trials and establishing networked collaborations with specialized centers, facilitating patient referrals, and ensuring that advanced treatments, like CAR-T cells or bispecific antibody therapies, reach those who need them most.

Our findings align with the global trend of improving survival in MM patients, emphasizing the role of advanced therapies and healthcare resource availability. For instance, our median OS of 72.5 months (2008–2015) could be comparable to the 86.7 months reported by López-Muñoz et al. (2005–2019), although the time periods are not exactly the same. Notably, the OS for our cohort C (2016–2022) has not yet been reached, indicating potentially superior outcomes. In comparison, Rodríguez-Lobato et al. reported a median OS of 61.2 months for their 2000–2015 cohort, which parallels our results of 50.7 months for 2000–2007 and 72.5 months for 2008–2015. Additionally, a sub-analysis of survival by decades, inspired by Puertas et al. (2000–2020), revealed medians of 68.5 and 72.4 months in our study versus 61.8 and 103.6 months at Salamanca University Hospital. While the time periods examined are not identical, this difference likely reflects variations in access to clinical trials and therapies between institutions.

We observed a significantly longer OS in ASCT recipients compared with non-recipients (132.66 vs. 38.81 months), reflecting differences in baseline characteristics, as non-candidates are typically older and have more comorbidities. Survival improved notably across cohorts for ASCT patients (81.8 months in cohort A, 132.7 months in cohort B, and not yet reached in cohort C; *p* = 0.011) compared with non-ASCT patients (30.8, 34.2, and 49.3 months, respectively; *p* = 0.23). The greatest gains were seen in younger patients (<60 years), where survival rose from 87.65 months in the first decade to 125.37 months in the second, with the median not reached in the last period. Younger, healthier patients benefit from aggressive management and higher ASCT eligibility, consistent with published studies. However, our results also lead us to a reflection not previously mentioned in other studies. It is true that the gain in life years, in absolute terms, is greater in younger patients, but we must consider that the potential gain in terms of additional months of life in older patients is much smaller. Specifically, patients over 70 years of age in our cohort have seen their survival increase from 17 months to 49.2 months, which, although more modest than that of younger patients in absolute terms, represents nearly a threefold increase in survival compared with the first cohort. For this reason, we believe that the outcomes in these older patients should not be considered poor, especially when considering the limitations inherent to this patient group.

Our findings also reflect the transformative impact of therapeutic advancements. The use of PIs and IMiDs increased substantially over time, from minimal use in cohort A (2000–2007) to 68.4% (PIs) and 13.4% (IMiDs) in cohort B, and 86.6% and 70.1%, respectively, in cohort C. Moreover, anti-CD38 monoclonal antibodies were incorporated as part of first-line treatment for 20.1% of patients in cohort C, further driving OS improvements.

Despite these findings, one limitation of our study is the lack of data on second-line and subsequent therapies, which also influence long-term outcomes. Nevertheless, our focus on first-line induction therapy provides critical insights into the early management of MM. A major strength of our study lies in its long-term follow-up, leveraging comprehensive data from both electronic and paper records to minimize patient attrition, particularly from earlier periods. Furthermore, our results reflect real-world clinical practice, which often diverges from clinical trial settings but is crucial for understanding the broader impact of advancements in MM treatment.

In addition to exploring the impact of novel therapies, it will be important to assess their long-term benefits and cost-effectiveness, particularly in real-world settings beyond specialized centers. Research focused on preventable deaths and evaluating the true impact of these therapies on OS will help optimize treatment protocols. It is also crucial to explore how these therapies affect different patient populations, including older patients, and how to improve their accessibility in non-specialized centers. 

Finally, further studies are needed to understand the long-term quality of life and functional status of MM survivors, as extending survival with innovative treatments should also consider the impact on daily living and overall well-being. This area of research will be crucial for optimizing treatment strategies and ensuring a more holistic approach to patient care.

## 5. Conclusions

Our analysis demonstrates a significant improvement in OS over time across all age groups, with the most pronounced and statistically significant increases observed in younger patients, while the survival improvement is more modest among patients over 70 years old. Furthermore, OS was higher in patients who underwent autologous hematopoietic stem cell transplantation compared with those who were not eligible for transplantation. These findings support the notion that MM could now be considered a chronic disease and potentially curable in a subset of patients.

Our findings underscore the substantial survival gains achieved in MM over the past two decades, driven by therapeutic innovations and improvements in clinical practice. However, the persistent survival disparities between healthcare settings highlight the need for equitable access to advanced therapies and the continued optimization of MM management strategies.

Finally, we emphasize the critical importance of analyzing outcomes from routine clinical practice. Despite the inherent challenges, this approach is essential for ensuring that patients receive high-quality care and survival opportunities aligned with current medical knowledge.

## Figures and Tables

**Table 1:** Baseline characteristics of patients in each time period.

	Total (2000–2022)	Cohort A (2000–2007)	Cohort B (2008–2015)	Cohort C (2016–2022)	*p*-Value
Number of patients (%)	420 (100)	106 (25.2)	149 (35.5)	165 (39.3)	0.0013
Age at diagnosis, median (range)	64 (34.8–96.9)	61.4 (34.8–91.1)	65.2 (36.3–89.1)	65.4 (36.5–96.9)	0.368
Age at diagnosis,					
<60 years, *n* (%)	153 (36.4)	45 (42.5)	50 (33.5)	58 (35.4)	0.876
60–70 years, *n* (%)	124 (29.5)	31 (29.2)	46 (30.9)	47 (28)	0.143
>70 years, *n* (%)	143 (34.01)	30 (28.3)	53 (35.6)	60 (36.6)	0.006
Gender, male, *n* (%)	226 (53.8)	47 (43.9)	83 (55.7)	96 (58.5)	0.0002
Gender, female, *n* (%)	194 (46.2)	60 (56.1)	66 (44.3)	68 (41.5)	0.76
Ig isotype, *n* (%)					
IgG	250 (59.5)	65 (60.7)	86 (57.7)	99 (60.4)	0.029
IgA	94 (22.4)	17 (15.9)	41 (27.5)	36 (22)	0.006
IgM	2 (0.5)	1 (0.9)	1 (0.7)	-	-
IgD	4 (0.9)	-	2 (1.3)	2 (1.2)	-
Light chains only	53 (12.6)	13 (12.1)	14 (9.4)	26 (15.9)	0.052
Biclonal	2 (0.5)	-	1 (0.7)	1 (0.6)	-
Non-secretory	8 (1.9)	5 (4.7)	3 (2)	-	-
ASCT (%)	204 (48.6)	50 (47.1)	71 (47.7)	83 (50.3)	0.017

Abbreviations: Ig: immunoglobulin; ASCT: autologous stem cell transplant.

## Data Availability

Due to the sensitive nature of the data, information created during and/or analyzed during the current study is available from the corresponding author.
